# Service provider perceptions of transitioning from audio to video capability in a telehealth system: a qualitative evaluation

**DOI:** 10.1186/s12913-017-2514-7

**Published:** 2017-08-14

**Authors:** Robyn Clay-Williams, Melissa Baysari, Natalie Taylor, Dianne Zalitis, Andrew Georgiou, Maureen Robinson, Jeffrey Braithwaite, Johanna Westbrook

**Affiliations:** 1 Australian Institute of Health Innovation, Faculty of Medicine and Health Sciences, Macquarie University, Level 6, 75 Talavera Rd, Sydney, NSW 2109 Australia; 2 Healthdirect Australia, Level 19, 133 Castlereagh St, Sydney, NSW 2000 Australia

**Keywords:** Telehealth, Collaboration, Healthcare delivery, Decision support tools, Implementation

## Abstract

**Background:**

Telephone consultation and triage services are increasingly being used to deliver health advice. Availability of high speed internet services in remote areas allows healthcare providers to move from telephone to video telehealth services. Current approaches for assessing video services have limitations. This study aimed to identify the challenges for service providers associated with transitioning from audio to video technology.

**Methods:**

Using a mixed-method, qualitative approach, we observed training of service providers who were required to switch from telephone to video, and conducted pre- and post-training interviews with 15 service providers and their trainers on the challenges associated with transitioning to video. Two full days of simulation training were observed. Data were transcribed and analysed using an inductive approach; a modified constant comparative method was employed to identify common themes.

**Results:**

We found three broad categories of issues likely to affect implementation of the video service: social, professional, and technical. Within these categories, eight sub-themes were identified; they were: enhanced delivery of the health service, improved health advice for people living in remote areas, safety concerns, professional risks, poor uptake of video service, system design issues, use of simulation for system testing, and use of simulation for system training.

**Conclusions:**

This study identified a number of unexpected potential barriers to successful transition from telephone to the video system. Most prominent were technical and training issues, and personal safety concerns about transitioning from telephone to video media. Addressing identified issues prior to implementation of a new video telehealth system is likely to improve effectiveness and uptake.

**Electronic supplementary material:**

The online version of this article (doi:10.1186/s12913-017-2514-7) contains supplementary material, which is available to authorized users.

## Background

Emerging evidence demonstrates the contribution of telehealth to improved health outcomes and quality of life [1]. Multiple systematic reviews have examined the benefits and challenges of using telephone [2, 3] or internet based video technology [4–6] for the provision of telehealth services. With the advent of smartphones and rapid advances in internet availability in remote areas, many service providers who began their consultations using an audio-only telephone are now transitioning to video technology. However, there is limited information available on the challenges for providers of transitioning from a telephone to a video service. In particular, moving from audio to video technology will require changes in service provider behaviour, and changes in the way that information is made available to providers to assist them in decision-making about care during consultations.

Current approaches for evaluating video services have shortcomings, [7] particularly centred on healthcare professional practice adaptations [4]. Evaluations typically involve assessing one or more of: patient clinical outcomes, economic outcomes, and provider or patient satisfaction with the new service [8]. Where satisfaction measures are collected, survey tools are used [8] which provide limited in-depth information about user experiences and views. In addition, few formative assessments are conducted in natural settings, [7] despite evidence that an intervention’s success is dependent on contextual factors [9].

In view of these limitations, we undertook a formative evaluation of a telehealth service, comprising a combination of interviews with providers and observations of how the telehealth is delivered in a workplace setting with a simulated patient (actor). Assessing telehealth services as integrated systems, and involving typical users and real world situations as part of the evaluation, is one way to enable problems to be identified and then addressed [10]. Seeking the views of participants who have interacted with the newly integrated system will allow identification of factors that may influence decision-making, transition and implementation [11].

## Methods

### Aim

Our study aimed to identify staff perceptions of the potential barriers, benefits, and safety issues associated with transitioning from a telephone helpline to an audio-visual telehealth service. This study was part of a larger project, evaluating implementation of the new service, that included analysis of routinely collected service data.

### Context

The Pregnancy, Birth and Baby (PBB) helpline, a national phone and online service established in 2010, provides information, advice and counselling about pregnancy, childbirth and parenting in a child’s first year. It is a non-commercial, government funded health information service, operated by Healthdirect Australia. In addition to an online website (www.pregnancybirthbaby.org.au), two levels of direct client support are provided by the PBB service: (1) Customer Support Officers (CSOs) provide standardised advice on specific topics, including planning for pregnancy, foods to avoid when pregnant, and breastfeeding. This advice is pre-packaged into factsheets on each topic. When delivering advice over the telephone, the CSO reviews the applicable factsheet via a computer terminal and verbally delivers the content over the phone. (2) Professionally qualified and registered Counsellors provide psychological support and counselling. Advice is tailored to the needs of the individual client, but may include sensitive topics such as the decision to terminate a pregnancy.

In 2014, the PBB telephone service was expanded to include a real time video conferencing capability (VCC). The video service was viewed as potentially offering advantages over the telephone service, such as access to callers’ non-verbal cues and visual information, supporting the establishment of greater rapport and trust with service users [12–14]. The quality of healthcare delivery has been shown to improve with the availability of visual information during nurse consultations [15].

The VCC uses open source WebRTC standards for real time video, audio and data communication. In preparation for this new service, a number of simulation sessions were held to train service providers, and to evaluate the final system design.

### Study design

We adopted a mixed method, qualitative approach. This involved 1) observation of the training of telephone service staff members who were required to make the switch from audio-only to video, and 2) pre- and post-training interviews with staff members and their trainers on the challenges associated with transitioning to the new medium. Combining observations and interviews in this way, to obtain an understanding of how technology is implemented and used in practice, is a common and accepted method in the field of human factors and ergonomics [16, 17].

### Participants

The study population consisted of counsellors and CSOs, who had completed classroom training and were due to participate in simulations over the study period, along with the system trainers. Training consisted of one day in the classroom, followed by one day of simulation, where providers used the actual VCC system to process calls from simulated patients (actors). Recruitment was undertaken using a mixture of purposive homogenous (system trainers) and convenience (CSOs and counsellors) sampling strategies. Fifteen staff members were invited via email to participate in interviews and all agreed. A selection of CSO and counsellor interviewees was assigned to the pre- or post-training groups based on their availability on the nominated interview days. Participants did not meet the researchers prior to commencement of the study, and were not informed of researcher personal motivations associated with the research.

### Procedure – simulation sessions

**Fig. 1:**
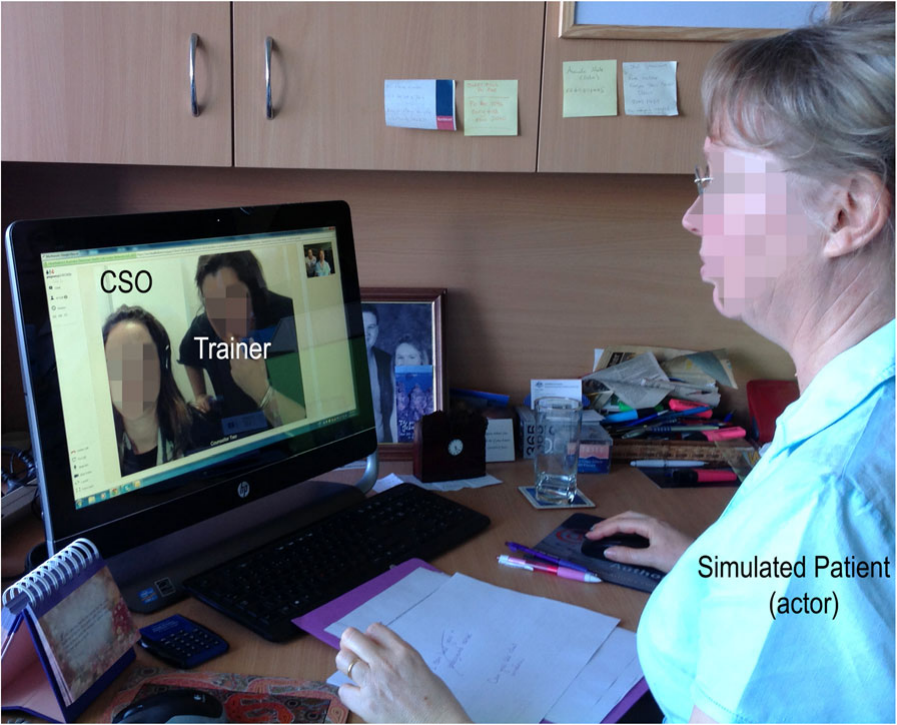
Observed simulation set-up from the simulated patient (actor) viewpoint

A scenario-focused training and evaluation process [18] was used: typical scenarios that might be encountered in the normal course of PBB service provision were developed and applied in training, then user performance was evaluated to test the interface. Providers used their normal workstation, which included a desk, telephone, and computer equipped with a clip on camera. Providers wore their normal telephone audio/mic headset, which was plugged into the computer headset port. Figure 1 shows the observed set-up from the simulated patient (actor) viewpoint. The CSO and Trainer can be seen on the screen, and appear as they would normally be presented to the patient. The simulated patient was located in a different city to the providers (approx. 950 km distant).

Simulations were set up to enable random calls to be made from the patient actor to the provider over a one day period. Providers answered video calls on demand, as part of their normal working day. All calls were initially answered by a CSO, then passed across to a counsellor if required by the situation.

Two full days of simulation training were observed by human factors trained researchers (MB, RCW): one day was observed from the patient actor viewpoint (two observers) and one day from the service provider viewpoint (one observer). Human factors is “the scientific discipline concerned with the understanding of interactions among humans and other elements of a system” [19]. The researchers used a simulation observer guide (Table 2), which focused on five domains, and made notes to capture additional issues that arose. Observers focused on participant behaviour and verbalisations while interacting with the equipment during the scenarios. Pre- and post-simulation interviews with participants were conducted to give the providers an opportunity to talk about the system design, the training and the VCC implementation process [11].

### Measures

Data collection during observations and interviews focused upon five domains: technical professional issues, organisational factors, system and work process effectiveness, and safety and quality. The nature of observation notes and interview questions associated with each domain are provided in Tables 1, 2 and 3.

**Table 1:** Simulation observer guide

Evaluation domain	Time	Description of data collected during observations
Demographics and background		Brief outline of the purpose of simulated call
Technical domain		How well did the system perform? Technical problems (e.g. the system was slow)? Were problems fixed? How? Areas for improvement?
Clinical/professional domain		Clinician/professional behaviours
Organisational domain		Real or potential negative effects of using the video service? (e.g. effect on ability to read and follow guidelines)
Effectiveness domain		Impact of the service on the healthcare system? Benefits the service makes to the work/performance? Benefits to consumer?
Safety and quality domain		How safe is this service? What risks did you pick up on? How can those risks be minimised or avoided?

**Table 2:** Pre-simulation interview questions with service providers

Demographics and background	Age, gender IT literacy Telehealth experience Previous training in telehealth
Clinical/professional domain	Do you think the video service will enhance the delivery of health advice, compared to the telephone service? Will the system improve service user outcomes? If so, how?
Organisational domain	Are there any real or potential negative effects of using the video service?
Effectiveness domain	What impact (if any) do you think the service will have on the healthcare system? What benefits do you expect this service to make to your work, and to consumers? How will you know if these benefits have been realised?
Safety and quality domain	How safe is this service? What are the risks of this service? How can those risks be minimised or averted?

**Table 3:** Post-simulation interview questions with service providers

Demographics and background	Age, gender IT literacy (Poor/Fair/Good /Excellent) Number of years telehealth experience
Technical domain	How well did the system perform during consultations? Did you experience any technical problems? Were the problems expected or unexpected? Were you able to fix these problems? How? Did you find the video service easy to use? Why/why not? How does it compare to the telephone service in terms of ease of use? How stressful was it using the video service? How does it compare to the telephone service in terms of stress levels? Can you think of any ways the video capability could be improved?
Training	Now that you’ve run through a few scenarios, do you think the training that you received was sufficient? Can you think of any areas where training or extra training is needed?
Safety and quality domain	Did you have any fears or reservations about using the video service? Have these been realised? Did using the video capability make it easier to communicate with callers? Were you able to get more information from callers? Do you think you were able to give better advice using video compared to the telephone? Do you have any other comments you’d like to make about the simulations or the video service in general?

### Analysis

Interviews were audio recorded and transcribed verbatim. Inductive thematic analysis of transcribed interviews and observation notes was undertaken to identify key themes across the broad domain areas relating to implementation of the video service. A modified constant comparative method [20] was used to facilitate identification of themes. This involved a first pass where each interview was coded by three researchers (RCW, MB and NT), and broad concepts developed and agreed. A second pass allowed for identification of sub-categories under the broad concepts. The researchers met and compared codes to resolve disagreements, and add new codes. Two researchers (MB and RCW) grouped codes together, and discussed implications, to arrive at themes.

## Results

Pre-simulation, four customer support officers (CSOs) and five counsellors participated in a semi-structured interview over the telephone. Following simulation, six post-simulation interviews were conducted with participants, three over the telephone and three face-to-face (two trainers, two CSOs and two counsellors). Demographic information about the participants, who were all female, is shown in Table 4.

**Table 4:** Participant demographic summary

	Age: Mean, Range (yrs)	Self rated computer literacy	Telehealth experience: Mean, Range (yrs)
Pre-simulation (*n* = 9)	30 (21–49)	Fair: 2 Good: 6 Excellent: 1	2 (0.5–5)
Post-simulation (*n* = 6)	34 (24–44)	Fair: 0 Good: 5 Excellent: 1	2.3 (1–3)

### Results of the thematic analysis

Interviews were conducted by three female PhD qualified professional research academics (MB, NT, RCW), with experience in qualitative research methods. Average pre-simulation interview length was 26.2 min, and 22 min post-simulation. Three broad categories emerged from an initial analysis of the interview data: social, professional, and technical issues. Within these three categories, eight sub-themes were identified; they were: enhanced delivery of the health service, improved health advice for people living in remote areas, safety concerns, professional risks, poor uptake of video service, system design issues, use of simulation for system testing, and use of simulation for system training.

Views of the video consultation service were consistent among participants, and data saturation was reached. Overall, providers were quick to identify potential problems with video implementation, and some participants struggled to identify any benefits of using the new service. This was particularly the case for CSOs:

“*I think for the medical lines [doctors, nurses, etc.], I think it’s really appropriate. I think it’s a brilliant idea … but I don’t think it’s actually appropriate for our line [counselling, non-medical advice, etc.]. It’s stressing a lot of the staff. There are several staff who are probably going to leave. There are staff who are going to, if they’re forced to do it, will probably leave. There are staff who are trying desperately to avoid it*.” (CSO 3)

### Enhanced delivery of the health service

Most participants felt the service would be of benefit to counsellors who provide medical advice to callers, but of less value to the CSOs who provide standardised advice. Some providers felt that being face-to-face with a caller would increase engagement with callers:

“*Any kind of visual aspect can potentially enhance the connection with the caller, rapport with the caller in the initial stages of a call*.” (Counsellor 5)

Some providers also felt that the video view would provide them with useful information about the caller and their situation, which might help them make better decisions about what information to provide:

“*I guess, being able to see a person might give you, you know, that greater kind of ability to being able to read their body language, and you know, seeing where they’re at, all those things that you’re kind of missing when you’re on the phone*.” (Counsellor 4)

### Improved health advice for people living in remote areas

A frequently cited benefit of the video service was that it would allow clients in remote and rural areas to have access to face-to-face advice. This was viewed as better than telephone advice, but not as effective as seeing someone in person:

“*I think it’s giving access to people who then can access medical services through rural or regional or even people who have disabilities or who are elderly, house bound, whatever, they don’t have to go their GP or go and see a nurse. They can call a service and still been seen. So I think that’s quite a positive thing for the future.*” (Counsellor 2)

### Safety concerns

Providers were apprehensive about being recorded during video consultations and recordings being posted on websites, and felt that this was likely to influence their decision about the type of health advice they provided. Providers felt exposed, and were uneasy about the prospect of callers using the video service inappropriately. They explained that, with the current telephone service, they occasionally received crank calls and expected that these would also be a problem on the video line. The difference was that “*once you see something it’s very hard to un-see*.” (CSO 2)

### Professional risks

Participants raised several concerns about the impact of the introduction of video on their professional roles. Participants believed that remaining professional both during and after a distressing call would be more difficult using the video service than the telephone service, because their emotions would be more visible on video. Providers recognised that video counselling required them to attain a “*brand new skill set*” (CSO 2).

Providers also believed that it would be more difficult to seek advice from a colleague during a video call, particularly if this involved leaving their desk. They explained that this might affect their decision to seek advice from colleagues:

“*I think a lot of people would feel like they can’t really ask for help all that much, particularly if someone is quite distressed, you don’t want to leave them*.” (CSO1)

Participants worried that callers might lose confidence in their ability to give advice if they were seen reading from factsheets, and that this might affect their decision making around the way in which factsheets are accessed and used. Similarly, several providers were apprehensive about their appearance leading callers to think that they were inexperienced:

“*If someone sees the counsellor at the other end they might think things like you look young, are you really experienced enough to kind of help me*” (Counsellor 5).

Providers believed that callers using the video service would expect visual demonstrations of practices or techniques. They recognised that one of the benefits of video is being able to show people how to do something, rather than just explain how to do it, but providers were quite anxious about this, because they were not trained to provide demonstrations:

“*With a video thing the first thing they’re going to say is, ‘Show me.’ There is no point in going through the hoops a caller has to go through in order to call in with a video unless they’re going to ask the question, ‘Show me.’ And the reality is I don’t have the training.*” (CSO3)

### Poor uptake of video service

All participants believed that uptake of the video service for the PBB line would be poor. This was mainly because they thought callers would prefer to remain anonymous. Many providers explained that the issues discussed with counsellors were often of a private or embarrassing nature. Obtaining advice while not being seen or identified was viewed to be one of the main reasons people used the telephone service.

“*I think that some people, we get a lot of calls particularly by people who are wanting to remain anonymous, so I think that in those instances it would be difficult for a person like that to use a video service because obviously that’s disclosing a lot more than a phone conversation does*.” (Counsellor 5)

Some providers felt that potential clients may struggle with the new technology. Others highlighted the inability of people to multi-task while on a video call in comparison with the telephone, and also the extra time needed to connect to the video service:

“*I guess most of them – they’re just wanting to pick up a phone, they’re wanting answers pretty quickly whereas I think trying to connect online and go through a video calling and if there’s problems it’s just going to take, I guess, longer than what they’re expecting*.” (CSO 2)

### System design issues

Many technical problems were encountered during the simulations, including sound and video quality issues, and the inability to transfer calls from CSOs to counsellors. Technical problems affected how the CSOs and counsellors perceived the system:

*“… there seemed to be a lot going on, where it wasn’t working very well … I didn’t feel very good about it at all … it also gets really quite frustrating* …” (CSO 3)

System design issues were also identified, including camera positioning problems, difficulty with the logon protocols, technical issues associated with transferring calls from one provider to another, difficulty with quickly locating decision support tools while on a call, and the absence of effective aural alerts to incoming calls.

### Use of simulation for system testing

The training was generally considered beneficial, however some negative effects were observed as a result of training on an immature system, and interruptions to repair technical faults were frequent. These disruptions were perceived by participants to have impacted the effectiveness of training, with the requirement to improvise to work around technical issues negating the benefit of training for some participants:

“*[because of the poor sound quality] I couldn’t really understand [what the client was saying] … I knew it was a transfer [from CSO to counselor] … that was me … absolutely winging it”* (CSO 4)

### Use of simulation for user training

Both the researchers and participants felt that additional practice ‘being on video’ would be beneficial, including training on how to position the video camera, and how to achieve ‘video presence’ including the importance of non-verbal communication and ‘where to look’:

*“ …the things that were a little bit difficult … was knowing exactly where to look [in terms of the camera] … I’m not sure if it was perceived as looking at [the client] or away.”* (Counsellor 5)

The need for additional training was identified in pre-session system set-up and testing, particularly microphone and speaker testing, and screen customisation. Most of the participants felt that the training was adequate, but expressed the preference for more practice in realistic environments before going live.

## Discussion

The issues identified are likely to affect successful implementation of the video service, and make it difficult for providers to transition from telephone to video. While technical issues identified in the study can be addressed with design changes or additional training, the social and professional issues identified in this study are previously unreported, and require detailed consideration both in the training and establishment of these types of services. While much has been written about the safety of patients in regard to video telehealth, [21–23] there is little research on provider safety. Perhaps the question of provider safety -- in terms of psychological and physical safety -- did not arise in these previous studies, as preparation for a research project, including obtaining participant consent, means that client participants are normally already in an established therapeutic environment or otherwise vetted by the research team. In the PBB system that we evaluated, calls are ad hoc and clients are not vetted, hence service provider safety comes to the fore. Psychological safety may be at risk due to for example crank callers exposing themselves physically over the video link; physical safety may be at risk due to for instance a client posting a screenshot of a counsellor who advises on pregnancy termination on an online public forum. For provision of on-demand video telehealth, such as the PBB, strategies to safeguard staff must be developed, tested and implemented. Providers must be informed of the existence of these safeguards, to alleviate the concerns this work has uncovered.

Our study results were fed back to the service provider organisation, and many changes were made to address the issues identified. These changes included that, when CSOs made initial contact with the patient, the video link was not immediately activated. In this way, CSOs could safely screen potential clients via audio, and decide whether to pursue the call, before transferring them to the counsellors who were on video. Another modification was the inclusion of black screens behind service providers, rather than a view of the provider’s workspace, to minimize extraneous information presented to clients.

In addition to identifying issues with transitioning to the new system, our study highlighted the importance of training providers in how to communicate in a video-based system. While simulation is an accepted method for training in healthcare, including training in non-technical skills such as communication between clinicians and patients, [24] training clinicians in interview techniques more typically involves workshops and face-to-face role play [25]. Therefore, introducing the use of video as part of a training programme is a new consideration. A recent systematic review on training methods for telehealth providers found gaps in research and current knowledge in relation to the importance of provision of hands-on training opportunities, such as simulation [26]. Our study adds to the evidence in favour of simulation as a telehealth training method.

When training providers in video-based communication, ‘video presence’ should be one of the skills to consider. Training in ‘video presence’ might involve discussing the importance of non-verbal communication (perhaps led by the counsellors) followed by instruction on camera set-up and ‘where to look’. Experts recommend that speech be slowed and body language, such as hand gestures, be augmented for effective video counselling (particularly so if the client is using a smaller screen) [21]. These do not come naturally, and should be practised. Participants might then practice role-plays with each other on a facility where they can self-observe, such as Skype. Sessions could be recorded and played back to the participants to provide immediate feedback on their performance. At least some of the training should involve demonstrations of how to create a video presence, practice in delivering content over a video link, feedback about the strengths and weaknesses of their delivery, and re-practice the delivery on actual (functioning) equipment.

While some technical problems can be addressed in a straightforward manner via a combination of system redesign and training, this is not always the case. International experience with provision of video telehealth services has identified technological problem-solving as a major concern and a possible limitation on future practice [21, 22]. By affecting how the CSOs and counsellors perceive the system, technical problems have the potential to erode trust in the technology. In planning for implementation of the PBB service, given that providers were accustomed to using computers as part of the telephone telehealth service, there was an implicit assumption that they would have sufficient technical competency to operate the video system. The changes in workflow and decision making required to use the video system, however, made the transition more complicated than expected.

It is critical for effective provision of care that technology is simplified so that the right information is presented to the provider at the right time [27]. Decision making for the CSOs would have been easier and quicker, for example, had the decision support tools been more easily accessible. Rather than requiring a high level of computer literacy, organisations might benefit from closer collaboration with providers during the final stages of development [10] to enable prioritisation of system information and ‘declutter’ modes. System complexity has been identified as a contributor to patient safety risk [22].

### Limitations

The CSOs and counsellors who participated in the simulation and associated interviews were not the same CSOs and counsellors who were interviewed pre-simulation. Therefore, we were not able to assess the degree to which simulation training changed perspectives of individuals. All participants were female, however there we no male CSOs or counsellors on staff at the time of the study. While we had a small number of participants, we did reach data saturation in the later interviews. In addition, the test of the system and the training provided were part of the same exercise. This made it difficult to extricate the true effects of the training, as trainees dealt with technical problems as they practised using the system. Organisations might consider separating system testing from provider training, as training with a system that is not the final design or not working correctly is likely to have a negative effect on competency and foster distrust of the equipment.

## Conclusion

In exploring service provider experience of planning for the transition from audio to video capability in a telehealth system, we identified a number of unexpected potential barriers to successful transition to the video system. In particular, technical and training issues, and personal safety concerns about transitioning from telephone to video media, are likely to hamper implementation of the new system, and adversely affect effectiveness of the service. Addressing these issues is likely to improve effectiveness and uptake.

## Additional file

Additional file 1: Themes and associated quotes. (DOCX 18 kb)

## Abbreviations

CSO: Customer Support Officer
GP: General Practitioner
PBB: Pregnancy, Birth and Baby
VCC: Video Conferencing Capability
