# Supplementary material for: Service provider perceptions of transitioning from audio to video capability in a telehealth system: a qualitative evaluation
Source: BMC Health Serv Res. 2017 Aug 14;17:558. doi: 10.1186/s12913-017-2514-7 (PMC5557607; doi:10.1186/s12913-017-2514-7)
Supplement: Additional file 1: — Themes and associated quotes. (DOCX 18 kb) [file 12913_2017_2514_MOESM1_ESM.docx]

**Additional file 1: Themes and associated quotes**

| **Category** | **Theme** | **Quotes** |
| --- | --- | --- |
| **Social** | Enhanced delivery of the health service | “*Well, I guess, you know, anytime I have called for myself, I’ve called a nurse, you know, with my children it just would be so much better for the nurse to be able to actually see that lump on the top of the child’s head instead of trying to describe it, in terms of a 50 cent coin, or you know, a 10 cent coin, yeah, that’s where it would really be beneficial, to make a proper diagnosis I guess*.”  (Counsellor 4)  “*If we were medical I'd say absolutely, brilliant idea, they can hold up a bub and go, ‘This is the rash that my bub has got’, and they go, ‘Oh, yes, that is milia. That is –‘ whatever it is. I think it's brilliant for medical stuff. But we're not a medical line.*”  (CSO 3)  “*Any kind of visual aspect can potentially enhance the connection with*  *the caller, rapport with the caller in the initial stages of a call*.”  (Counsellor 5)  “*Well, I guess it would, it’s more comforting, I think, and I guess reassuring to actually be able to see someone while you’re talking with them and get what they’re saying. You can always do hand gestures and things like that which, I guess, express things more.*”  (CSO4)  “*I guess, being able to see a person might give you, you know, that greater kind of ability to being able to read their body language, and you know, seeing where they’re at, all those things that you’re kind of missing when you’re on the phone*.” (Counsellor 4) |
|  | Improved health advice for people living in remote areas | “*I think it’s giving access to people who then can access medical services through rural or regional or even people who have disabilities or who are elderly, house bound, whatever, they don’t have to go their GP or go and see a nurse. They can call a service and still been seen. So I think that’s quite a positive thing for the future.*”  (Counsellor 2)  “*I think that the main impact of that is, as I said with those living in rural locations being able to access information much more readily from their homes, which it’s hard enough to get into a service in a rural area let alone to be able to access it at all sometimes.*”  (Counsellor 5) |
| **Professional** | Safety concerns | “*once you see something it’s very hard to un-see*.” (CSO 2) |
|  | Professional risks | “*I think that the main concern is having lots of things going at once while you’re also trying to – obviously, to keep maintaining some eye contact with the person you’re speaking to*.” (Counsellor3)  “*I think a lot of people would feel like they can't really ask for help all that much, particularly if someone is quite distressed, you don't want to leave them. Whereas on the phone because they can't see you, I don’t know, there's just a level of intimacy that's missing and it's okay to place them on hold and ask the question then come back because if anything they know that you've gone away and done something for them and they’re okay with that*.”  (CSO1)  “*And perhaps having to be just a little bit more self-reliant and not rely so much on the information that you sometimes have. Or I suppose it would just be a matter of communicating to the person, you know bear with me while I find the right number for you or just explaining to them what you're doing in that moment*.”  (Counsellor 5)  “*If someone sees the counsellor at the other end they might think things like you look young, are you really experienced enough to kind of help me*”  (Counsellor 5).  “*As well as if it’s someone quite older who obviously – I’m quite young and haven’t had children or anything and they would be speaking to me. And they might think that I don’t know what I’m talking about because, yeah, I don’t have the firsthand experience*.”  (CSO 4)  “*With a video thing the first thing they're going to say is, ‘Show me.’ There is no point in going through the hoops a caller has to go through in order to call in with a video unless they're going to ask the question, ‘Show me.’ And the reality is I don't have the training. I've got – I don't have kids. I've got no idea how to burp a baby. I have literally zero idea how to burp a baby. I can't even say, ‘Okay, you need to wrap a baby this way or you need to – when you're doing it, do this way. Oh no, if you hold him this way you'll find that's more effective.’ I can't answer that*.”  (CSO3) |
|  | Poor uptake of video service | “*I think that some people, we get a lot of calls particularly by people who are wanting to remain anonymous, so I think that in those instances it would be difficult for a person like that to use a video service because obviously that’s disclosing a lot more than a phone conversation does*.”  (Counsellor 5)  “*But again, it's all about the availability of the technologies with the callers as well...they've got to be sitting in front of the computer or a laptop or something that has that camera capability and I just honestly have no idea how many people actually have that*.”  (CSO1)  “*I guess most of them – they’re just wanting to pick up a phone, they’re wanting answers pretty quickly whereas I think trying to connect online and go through a video calling and if there’s problems it’s just going to take, I guess, longer than what they’re expecting or anticipating the service to be. So I think that in terms of, yeah, timing, I don’t think it would be suitable*.”  (CSO 2) |
| **Technical** | System design issues | “… *there seemed to be a lot going on, where it wasn’t working very well … I didn’t feel very good about it at all … it also gets really quite frustrating* …” (CSO 3)  *“… well there’s 50 different things that I can change in the control panel … they [providers] are technically savvy but I think this is also taking it to a whole other level of being technically savvy … there needs to be an expert level*.”  (Trainer 1) |
|  | Use of simulation for system testing | “*[because of the poor sound quality] I couldn’t really understand [what the client was saying] … I knew it was a transfer [from CSO to counselor] … that was me … absolutely winging it”*  (CSO 4) |
|  | Use of simulation for user training | *“ …the things that were a little bit difficult … was knowing exactly where to look [in terms of the camera] … I’m not sure if it was perceived as looking at [the client] or away.”*  (Counsellor 5)  *“I’ve struggled with [set-up] because these workstations aren’t set up like our normal ones, they don’t have all the resources at hand.*”  (CSO 4)  “*… [it would be useful to train in] that environment where you’re knowing that there is a back-up [of calls] or [where] you are obligated to answer a few calls one after the other”*  *(Counsellor 5)* |
